# The controversial interplay between hyperinflammation and immunosenescence in elderly critically ill co-infected patients

**DOI:** 10.1186/s12877-026-07767-2

**Published:** 2026-06-05

**Authors:** Wenwen Sun, Yajie Zhao, Xin Wan, Jie Fang, Zhihong Xu

**Affiliations:** 1 Department of Geriatric Medicine, Ruijin Hospital, Shanghai Jiao Tong University School of Medicine, 197 Ruijin 2nd Road, Shanghai, 200025 China; 2 Shanghai Institute of Immunology, Shanghai Jiao Tong University School of Medicine, Shanghai, China; 3 Department of Information Processing Centre, Ruijin Hospital, Shanghai Jiao Tong University School of Medicine, Shanghai, China; 4 Department of Pharmacy, Ruijin Hospital, Shanghai Jiao Tong University School of Medicine, Shanghai, China

**Keywords:** Immunosenescence, Hyperinflammation, Mortality prediction model, Critically ill, Geriatric, Co-infection

## Abstract

**Background:**

Aging involves both immunosenescence and chronic low-grade inflammation, which can escalate into hyperinflammation during acute infection. However, how the opposing forces of immunosenescence and hyperinflammation jointly determine mortality, and whether viral-associated triple infection exacerbates this imbalance, remains poorly understood. This study investigates these two questions in critically ill co-infected patients by examining the interplay between hyperinflammation and lymphopenia in fatal outcomes.

**Methods:**

This retrospective cohort study enrolled critically ill co-infected patients admitted hospital from February 2023 to September 2025, following rigorous inclusion and exclusion criteria to ensure data quality. Elastic net‑regularized logistic regression with 10‑fold stratified cross‑validation was applied to candidate variables prescreened by univariate logistic regression (*p* < 0.05); variables with non‑zero coefficients were then combined with the forced confounders (patient age and viral-associated triple infection) and refitted in a standard logistic regression model for unbiased estimation. Internal validation was then performed using 1000 bootstrap resamples. Model performance was evaluated via ROC curve, calibration curve, and predicted probability distribution plot. To better understand total protein (TP) and neutrophil-to-lymphocyte ratio (NLR) for mortality prediction, the individual TP, neutrophil, and lymphocyte counts of all patients were examined. To investigate whether viral-associated triple infection exacerbates immune dysfunction, we compared clinical and laboratory variables between patients with triple infection and those with non-viral double infection. Age-stratified analysis was also performed.

**Results:**

A total of 118 critically ill patients with bacterial-fungal co-infection were enrolled (mean age 68.6 years; 83% aged ≥ 60 years). Elastic net-regularized logistic regression (α = 0.3, λ = 0.4723) identified TP as an independent protective factor (OR = 0.876, *P* = 0.0014) against mortality, and NLR as an independent risk factor (OR = 1.026, *P* = 0.0182) for mortality. The final prediction model, Logit(P1) = 2.964 − 0.132×TP + 0.025×NLR + 0.035×patient age + 0.446×viral-associated triple infection, demonstrated promising discrimination (apparent AUC: 0.863 (95% CI: 0.786–0.94); optimism-corrected AUC: 0.836 (95% CI: 0.808–0.878); distribution overlap = 6.3%) and high calibration accuracy (Bootstrap-corrected calibration intercept=-0.176, slope = 0.801, mean absolute error = 0.0007). Among the 23 deceased patients, 87% had profound lymphopenia, while 74% exhibited neutrophilia. Additionally, 70% of patients had protein levels below 60 g/L, with 9 falling below 50 g/L. Compared to those non-viral double infection patients (*n* = 39), patients with viral-associated triple infection (*n* = 79) exhibited a more pronounced hyperinflammation-lymphopenia imbalance, with higher NLR, neutrophils, PCT, and CRP, along with lower lymphocytes, T-cell subsets, TP, and prealbumin. These findings were consistent in the age‑stratified subgroup of patients aged < 70 years (all FDR-adjusted q < 0.05).

**Conclusion:**

This study reveals immune dysregulation characterized by hyperinflammation-immunosenescence coexistence; a preliminary prediction model (TP, NLR, age, viral coinfection) requires prospective multicenter validation.

**Supplementary Information:**

The online version contains supplementary material available at 10.1186/s12877-026-07767-2.

## Background

Geriatric patients with severe co-infections represent a high-risk population, exhibiting substantially higher mortality than younger individuals [1, 2]. Aging is accompanied by profound remodeling of the immune system, termed immunosenescence, characterized by a decline in naive T cells, accumulation of memory cells, and impaired antigen presentation, leading to increased susceptibility to novel pathogens and reduced vaccine efficacy [3, 4]. Concurrently, the elderly often maintain a state of chronic low-grade inflammation known as “inflammaging”, which can escalate into uncontrolled hyperinflammation during acute infections, contributing to tissue damage and multiple organ failure [5–7]. These seemingly contradictory processes, immunosenescence and hyperinflammation, coexist in critically ill older patients [8, 9]. However, how the opposing forces of immunosenescence and hyperinflammation jointly determine mortality remains poorly understood.

Coinfection, such as influenza complicated by bacterial and fungal pneumonia or SARS-CoV-2 co-infected with other respiratory viruses, is not uncommon in elderly patients with severe infection [10, 11]. Co-infection may further perturb immune homeostasis: persistent stimulation by multiple pathogens could accelerate T-cell exhaustion (exacerbating immunosenescence), while synergistic interactions between viruses and bacteria might provoke an even more intense inflammatory response [12]. Although co-infection has been associated with worse prognosis, direct evidence on whether it amplifies the imbalance between immunosenescence and hyperinflammation to increase mortality is lacking.

Therefore, the present study was designed to comprehensively evaluate the joint impact of immunosenescence (e.g., lymphocyte subsets, T-cell functional markers) and hyperinflammation (e.g., cytokine levels, inflammatory biomarkers) on fatal outcomes in a cohort of critically ill geriatric patients with multiple infection. The primary aim was to investigate how the opposing forces of hyperinflammation and immunosenescence jointly determine mortality. The secondary aim was to assess whether the presence of viral co-infection exacerbates this immunological imbalance. Our findings may deepen the understanding of immune pathophysiology in elderly patients with severe multiple infection and inform risk stratification and therapeutic strategies.

## Methods

### Study subjects and design

This retrospective cohort study was conducted at Ruijin Hospital between February 27, 2023 and September 12, 2025. Critically ill patients with targeted next-generation sequencing (tNGS) confirmed infection were screened for eligibility. Critically ill was defined as the presence of at least one of the following: ICU admission, septic shock, invasive mechanical ventilation, or acute respiratory failure (PaO₂/FiO₂ ≤300). Samples included bronchoalveolar lavage fluid, sputum, blood, urine, pleural effusion, peritoneal fluid, and catheter tips.

To ensure data integrity and homogeneity of the study population, a stepwise exclusion process was applied. Patients were first excluded if lymphocyte subset testing was not performed. Further exclusions were as follows: hematologic disorders (acute and chronic leukemia, lymphoma, myelodysplastic syndrome, multiple myeloma, aplastic anemia, and paroxysmal nocturnal hemoglobinuria), rheumatic and autoimmune diseases (systemic lupus erythematosus, moderate-to-severe rheumatoid arthritis, vasculitis, and Behçet’s disease), severe renal disease (chronic kidney disease stage ≥ 3, focal segmental glomerulosclerosis, membranous nephropathy, and immunoglobulin A nephropathy), solid tumors (≥ 3 stage), history of solid organ or hematopoietic stem cell transplantation, enrollment in drug trials, or major trauma (injury severity score > 15 or requiring surgical intervention within 48 h). Patients with missing data were excluded from the analysis. These criteria were designed to minimize confounding from pre-existing immunosuppression or non-infectious drivers of systemic inflammation.

The study was conducted in accordance with the Declaration of Helsinki and approved by the Ruijin Hospital Ethics Committee. Written informed consent was obtained from the patient whenever possible. For patients who were unable to provide consent due to delirium, sedation, or cognitive impairment, consent was obtained from their legal guardians or first-degree relatives according to the regulations of the ethics committee.

### Study variables

Medical records were reviewed to extract patient demographics, diagnoses, treatments, length of stay, survival status, hematologic parameters, imaging findings, and pathogenic test results. In this study, “viral-associated triple infection” (triple infection or viral coinfection) was defined as the simultaneous presence of viral, fungal, and bacterial pathogens. “Non-viral double infection” (double infection) was defined as the presence of fungal and bacterial pathogens only. The primary endpoint was in‑hospital mortality. Hospital stay length (HSL) was a secondary descriptive variable; it was not used in multivariable logistic regression because its distribution was heavily skewed and no clinically justified cutoff existed. Analyzed blood markers focused on key immune, inflammatory and nutritional parameters, including T lymphocytes, neutrophils, C-reactive protein (CRP), procalcitonin (PCT), the neutrophil-to-lymphocyte ratio (NLR), total protein(TP), pre-albumin, red blood cell (RBC) and hemoglobin (Hb). For patients undergoing bacterial and fungal testing, data on T-cell subsets (CD3^+^CD4^+^, CD3^+^CD8^+^) and viral infection detection were also required. All laboratory parameters were measured within the first 48 h after admission.

### tNGS (targeted next-generation sequencing)

tNGS served as a high-throughput, cost-effective diagnostic tool capable of simultaneously detecting a broad spectrum of pathogens (bacteria, fungi, and viruses), antimicrobial resistance (AMR) genes, and virulence factors. Key detectable viruses included severe acute respiratory syndrome coronavirus 2 (SARS-CoV-2), Epstein-Barr virus (EBV), cytomegalovirus (CMV), herpes simplex virus (HSV), respiratory syncytial virus A/B (RSV A/B), influenza virus A/B (Flu A/B), and parainfluenza virus 1/2/3 (PIV 1/2/3). This technique initially amplified target genes by using one or more specific primers, and then conducted high-throughput next-generation sequencing (NGS).

tNGS assay simultaneously detects bacteria, fungi, and viruses, and together with clinical symptoms, bacterial culture, and fungal GM/G tests, it enables the identification of co-infection or polymicrobial infection. The accuracy of tNGS significantly exceeded that of conventional microbiological tests (CMTs), and the cost of tNGS was much lower than that of metagenomic next-generation sequencing (mNGS) [13].

### Statistical analysis

Statistical analyses were performed in R. A two-tailed P-value < 0.05 was considered statistically significant. The data included continuous, categorical, and count variables.

The logistic regression analysis workflow proceeded as follows. Candidate variables were first screened using univariate logistic regression (*p* < 0.05). Then, Elastic Net with 10-fold stratified cross-validation was applied to the selected candidate variables. All continuous predictors were standardised to mean 0 and standard deviation 1 before applying elastic net. A grid search was performed over alpha (α) values of 0.1, 0.3, 0.5, 0.7, and 0.9. The α that maximized the cross-validated AUC was 0.3. For this α, λ was selected using the one-standard-error rule: the largest λ with cross-validated deviance within one standard error of the minimum deviance (λ1se) was chosen to favor model parsimony. Variables with non-zero coefficients from the elastic net were combined with the forced confounders (patient age and viral coinfection) finally refitted in a standard logistic regression model (SLRM) without penalisation using the glm function for unbiased estimation. Variance inflation factors (VIFs) were computed; any variable with VIF > 10 was removed, and the SLRM was refitted again. Internal validation was then performed using 1000 bootstrap resamples. The analyses were performed using the R packages “car”, “glmnet”, “pROC”, “rms”, “BootValidation”, “caret”, “regplot”, “ggplot2”, “ResourceSelection” and “dplyr”.

The discriminative ability and calibration accuracy of the final models were evaluated using the Area Under the Curve (AUC), predicted probability distribution plots (parameters of interest: difference, overlap), and calibration curves (mean absolute error, MAE). To further elucidate the role of TP-NLR in mortality prediction, an analysis was conducted on the individual TP, neutrophil, and lymphocyte counts of patients.

To address whether viral-associated triple infection exacerbates the hyperinflammation-immunosenescence imbalance, baseline demographic, clinical, and laboratory variables were compared between viral-associated triple infection and non-viral double infection groups. Following the overall analysis, age-stratified comparisons between the two groups were conducted. Normality for continuous variables was assessed using the Shapiro-Wilk test. Normally distributed variables are expressed as mean ± SD (standard deviation) and compared with the independent t-test. Non-normal continuous variables are summarized as median (IQR) and compared with the Mann-Whitney U test (including the group comparisons of HSL); categorical variables were presented as frequencies and compared using the chi-square test or Fisher’s exact test, as appropriate. P values < 0.05 were considered significant, and all tests were 2-tailed. P-values were adjusted for multiple comparisons using the false discovery rate (FDR, q < 0.05).

## Results

### General characteristics of enrolled patients

Following the application of strict exclusion criteria, 118 patients were analyzed, all of whom received guideline-directed antibiotic treatment. The mean age of the cohort was 68.6 years; 83% of patients were aged ≥60 years, and 63.5% were male. Most (92%) had pulmonary infections; 54% were hospitalized for ≥14 days, and mortality was 19.5%. 39 had bacterial-fungal (double) co-infection, while 79 had bacterial-fungal-viral (triple) co-infection.

### Results of univariate logistic regression analysis for mortality prediction

In univariate logistic regression analysis, 15 of the 27 examined variables showed a significant association with mortality (*p* < 0.05); odds ratios (ORs) with 95% confidence intervals (CIs) are provided in Table 1.

**Table 1:** The univariate and multivariate logistics analysis for mortality. Key predictors of mortality

Univirate logistics analysis				
Variables		OR	95%CI	*P*-Value
CD3 absolute count ( /U)		0.998	[0.996, 0.999]	0.0019
CD4 absolute count ( /U)		0.995	[0.991, 0.998]	0.0014
CD8 absolute count ( /U)		0.997	[0.994, 0.999]	0.0277
CRP (mg/L)		1.006	[1.000, 1.013]	0.0526
D-dimer (mg/L)		1.300	[0.690, 2.411]	0.4054
WBC count (10^9/L)		1.088	[1.027, 1.160]	0.0056
Lymphocyte count (10^9/L)		0.258	[0.087, 0.623]	0.0066
Neutrophil count (10^9/L)		1.099	[1.037, 1.174]	0.0023
RBC count (10^12/L)		0.601	[0.336, 1.032]	0.0716
Hb (g/L)		0.985	[0.966, 1.003]	0.1049
Platelet count (10^9/L)		0.991	[0.985, 0.996]	0.0021
eGFR ( ml/min/1.73m2)		0.979	[0.963, 0.995]	0.0121
Creatinine (umol/L)		1.004	[0.999, 1.009]	0.1314
BUN (mmol/L)		1.049	[1.001, 1.100]	0.0421
Uric acid (umol/L)		1.002	[0.998, 1.006]	0.2984
TBIL (umol/L)		1.003	[0.990, 1.012]	0.6118
TP (g/L)		0.861	[0.797, 0.919]	< 0.001
Prealbumin (mg/L)		0.987	[0.979, 0.995]	0.0014
ALT (IU/L)		0.936	[0.874, 0.994]	0.0418
AST (IU/L)		1.007	[0.960, 1.049]	0.7456
PCT (ng/MI)		1.028	[0.990, 1.075]	0.1492
NLR		1.036	[1.015, 1.062]	0.0031
Patient age (year)		1.026	[0.990, 1.070]	0.1876
Viral-associated triple infection		4.068	[1.28, 18.129]	0.0320
Pleural effusion		1.722	[0.679, 4.352]	0.2472
Lung lobes involved		4.614	[1.28, 30.036]	0.0479
Patient gender		0.553	[0.186, 1.467]	0.2544

Multivariates logistics analysis				
Variables	Coefficient	OR	95%CI	*P*-Value
Intercept	2.964	19.368	[0.059, 6913.064]	0.3113
TP (g/L)	-0.132	0.876	[0.802, 0.945]	0.0014
NLR	0.025	1.026	[1.008, 1.051]	0.0182
Patient age	0.035	1.035	[ 0.984, 1.099]	0.2191
Viral-associated triple infection	0.446	1.561	[0.364, 8.411]	0.5658

Univariate analyses for mortality and hospital stay length identified multiple associated variables. Elastic net-regularized logistic regression was applied. TP and NLR were selected, and patient age and viral‑associated triple infection were added as primary confounding factors

*Abbreviations:* *NLR *neutrophil-to-lymphocyte ratio, *Hb *Hemoglobin, *eGFR *estimated glomerular filtration rate, *TBIL *total bilirubin, *TP *Total protein, *CRP *C-reactive protein, *PCT *Procalcitonin, *BUN *Blood Urea Nitrogen

Variables, including CD3^+^ T cell count, CD3^+^CD4^+^ T cell count, CD3^+^CD8^+^ T cell count, lymphocyte count, TP, eGFR, platelet count, and prealbumin had ORs less than 1, suggesting that higher values may be associated with a decreased odds of death. Lymphocyte count showed the strongest protective effect (OR = 0.258, *p* = 0.0066). TP was the most statistically significant finding (*p* < 0.001) and demonstrated a consistent protective effect (OR = 0.861, 95% CI: 0.797–0.919).

ORs for lung lobes involved, viral infection, WBC, neutrophils, NLR, and BUN all exceeded 1, indicating a potential increased mortality risk with higher values. Among these, lung lobes involved had the strongest risk estimate (OR range: 1.27–27.17), though its wide confidence interval reflects considerable uncertainty.

### Final multivariable logistic model: TP, NLR, age, and viral-associated triple infection

Elastic net-regularized logistic regression was used to optimize both predictive performance and model interpretability.

TP and NLR were identified using Elastic Net-regularized logistic regression. The α that maximized the cross-validated AUC was 0.3, and λ was 0.4723. TP, NLR, and primary confounders (patient age and viral-associated triple infection) were selected to construct a final logistic regression model (Table 1). The model showed that TP has a coefficient of -0.132 (OR = 0.876, 95% CI: 0.802–0.945, *P* = 0.0014), indicating that for each one-unit increase, the odds of death decrease by approximately 12.4% on average. NLR has a coefficient of 0.025 (OR = 1.026, 95% CI:1.008–1.051, *P* = 0.0182), meaning that for each one-unit increase, the odds of death increase by approximately 2.5% on average. Although patient age (coefficient = 0.035, OR = 1.035, 95% CI: 0.984–1.099, *P* = 0.2191) and viral‑associated triple infection (coefficient = 0.446, OR = 1.561, 95% CI: 0.364–8.411, *P* = 0.5658) did not achieve statistical significance, they were included in the model as important confounding factors. The final prediction model for mortality is Logit(P1) = 2.964 − 0.132×TP + 0.025×NLR + 0.035×patient age + 0.446×viral-associated triple infection. The VIF for TP, NLR, patient age, and viral-associated triple infection were 1.084, 1.092, 1.150, and 1.038, respectively.

**Fig. 1:**
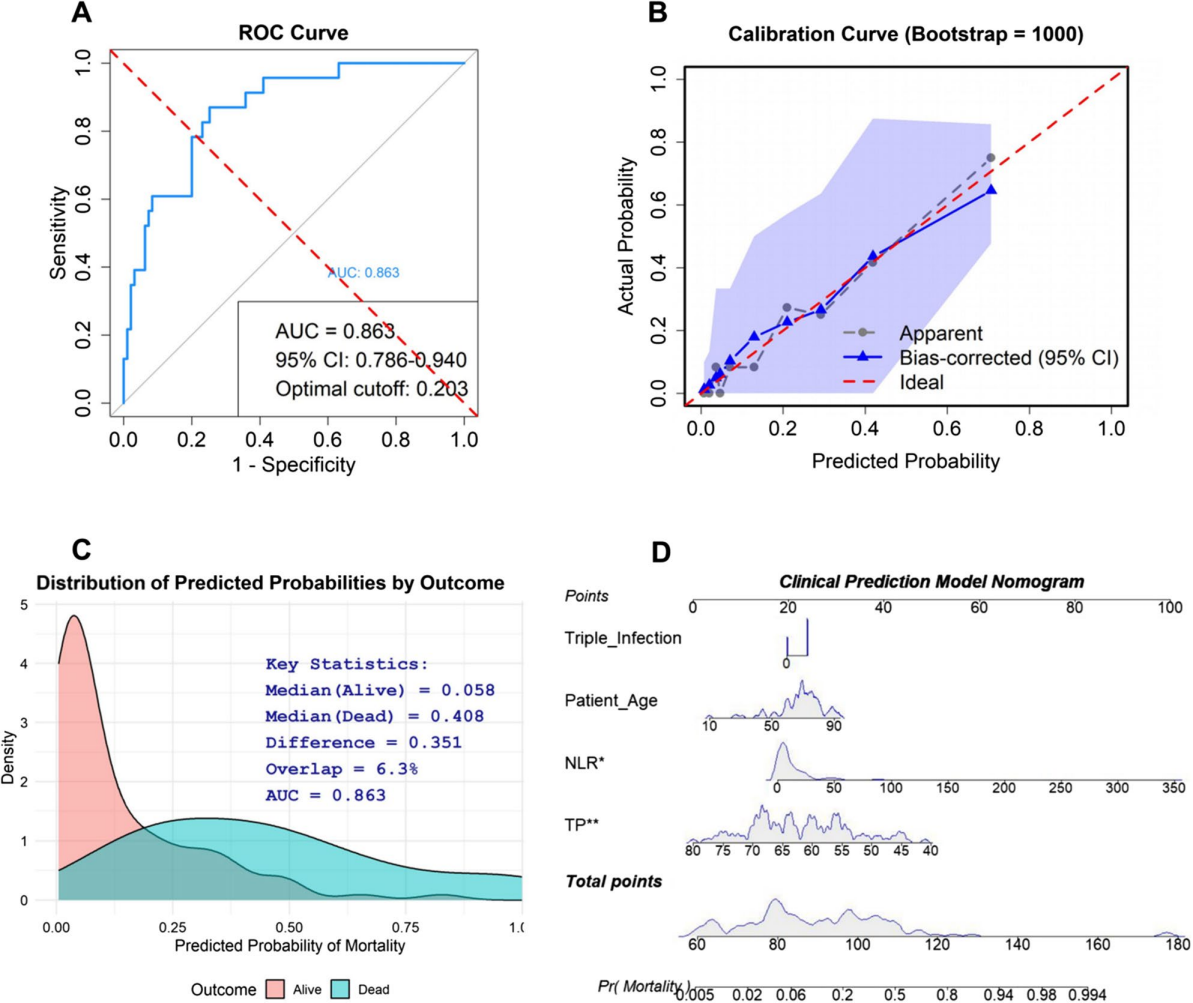
Performance evaluation of the mortality prediction models. **A** Apparent ROC curve (apparent AUC = 0.863). The bootstrap-corrected AUC was 0.836, which remains above 0.8, indicating good overall discriminatory ability for fatal and surviving cases after accounting for optimism. **B** Bootstrapcorrected calibration curve. Mean Absolute Error was 0.0007. The calibration intercept was -0.176 (95% CI: -0.804 to 0.751) and the calibration slope was 0.801(95% CI: 0.292 to 1.313), revealing reliable probability estimates. **C** In the predicted probability distribution plot, the small overlap (6.3%) and substantial difference (0.351) indicated that the model exhibits high confidence in its predictions for the vast majority of samples. **D** Nomogram. The intuitive graphical ruler of Logit(P1)=2.964-0.132×TP+0.025×NLR+0.035×patient age+0.446×viral-associated triple infection

The elastic net (λ_1se_) achieved a cross-validated AUC of 0.825. After adding primary confounders patient age and viral-associated triple infection to the model, the apparent AUC increased to 0.863 (95% CI: 0.786–0.94) (Fig. 1A). At the optimal cutoff of 0.203, accuracy, sensitivity, and specificity were 77.1%, 87%, and 74.7%, respectively, indicating good predictive efficacy. However, bootstrap internal validation (1000 resamples) revealed an optimism of 0.027, resulting in an optimism-corrected AUC of 0.836 (95% CI: 0.808–0.878). The Bootstrap-corrected calibration intercept was -0.176 (95% CI: -0.804 to 0.751) and the calibration slope was 0.801(95% CI: 0.292 to 1.313) (Fig. 1B). The MAE of bootstrap-corrected calibration was 0.0007, confirming reliable probability estimates (Fig. 1B). The predicted probability distributions showed minimal overlap (6.3%) and a clear separation (difference = 0.351), reflecting high prediction confidence for most samples (Fig. 1C). The nomogram allows for direct outcome visualization (Fig. 1D).

NLR was testified to be the independent risk factor for mortality. To be specific, it is worthwhile to observe the data of neutrophil and lymphocyte counts.

### Lymphopenia, neutrophilia, and hypoproteinemia in deceased versus surviving patients

**Fig. 2:**
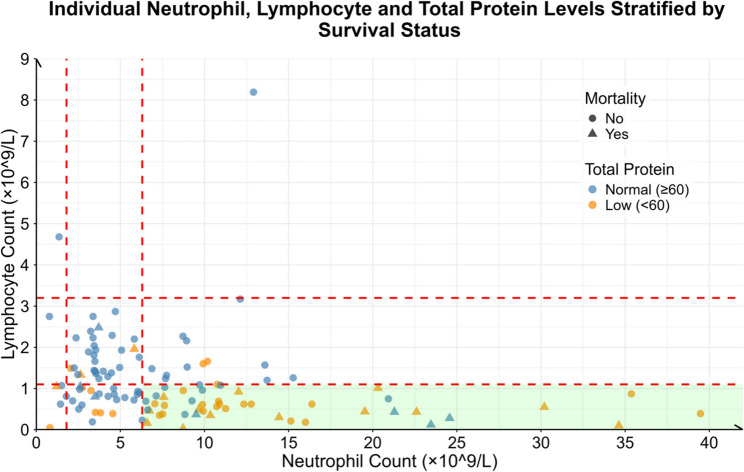
Levels of neutrophils, lymphocytes, and total protein of the enrolled patients. Among the deceased patients, 50% simultaneously had hypoalbuminemia (<60 g/L), lymphopenia, and high neutrophil levels, compared with only 18.95% of surviving patients. Moreover, 69.57% of deceased patients presented with both lymphopenia and neutrophilia, while this proportion was only 28.42% among survivors. Shapes indicate survival status: triangles (died) and circles (survived). Colors indicate albumin level: blue (albumin ≥60 g/L) and orange (albumin <60 g/L). The red dashed lines denote the normal ranges for neutrophil and lymphocyte counts

As shown in Fig. 2, among all enrolled patients, the simultaneous presence of hypoalbuminemia (< 60 g/L), lymphopenia, and elevated neutrophil counts was observed in 50% of deceased patients, compared with only 18.95% of surviving patients. Furthermore, 69.57% of deceased patients presented with both lymphopenia and neutrophilia, whereas this combination occurred in only 28.42% of survivors. In the deceased group, 16 out of 23 patients had neutrophilia, and 17 had hypoalbuminemia. In the survival group, 21 out of 95 patients had neutrophilia, and 28 had hypoalbuminemia.

These findings indicate that a dysregulated immune phenotype characterized by concurrent lymphopenia, neutrophilia, and low total protein is markedly more common in non-survivors.

### Viral-associated triple infection (virus, bacteria and fungus) exacerbates immunological dysregulation and hypoproteinemia

#### Overall analysis

Compared with the double infection group, the viral-associated triple infection group had significantly lower levels of CD3^+^ T cell (575 vs. 906 q = 0.0004), CD3^+^CD4^+^ T cells (248 vs. 49, q = 0.0001), CD3^+^CD8^+^ T cells (245 vs. 331, q = 0.0215), total lymphocytes (0.800 vs. 1.380, q = 0.0013), RBC (3.168 vs. 3.773, q = 0.0005), Hb (97.570 vs. 114.256, q = 0.0010), TP (q = 0.0005), and prealbumin (q = 0.0017). Conversely, significantly higher median values were observed for NLR (9.680 vs. 3.160, q = 0.0004), neutrophils (7.810 vs. 4.510, q = 0.0100), PCT (0.240 vs. 0.040, q = 0.0004), and CRP (q = 0.0215), and blood urea nitrogen (BUN, q = 0.0197) in the triple infection group. In addition, the triple infection group showed a greater number of lobar infiltrates, a significantly longer median hospital stay (19 vs. 11 days, q = 0.0071), and a higher mortality rate (25.5% vs. 7.6%, χ²=4.106, q = 0.0712) than those in the double infection group (Table 2).

**Table 2:** Statistical profile of immune dysregulation associated with polymicrobial co-infection. Descriptive and inferential statistics

Non-normally distributed continuous variables: statistical description and non-parametric tests (Mann-Whitney U analysis)				
Variables	Positive(median, interquartile range)	Negative (median, interquartile range)	*P*-value	p.FDR(q)
CD3 absolute count ( /U)	575.000 [295.000, 851.500]	906.000 [659.500, 1339.500]	< 0.0001	0.0004
CD4 absolute count ( /U)	248.000 [128.500, 402.500]	495.000 [315.500, 703.000]	< 0.0001	0.0001
CD8 absolute count ( /U)	245.000 [129.000, 372.500]	331.000 [221.000, 513.000]	0.0102	0.0215
CRP (mg/L)	54.000 [12.500, 97.300]	10.000 [5.500, 57.700]	0.0132	0.0215
D-dimer (mg/L)	1.060 [0.515, 1.640]	0.650 [0.400, 1.235]	0.0468	0.0635
WBC count (10^9/L)	9.270 [5.860, 12.685]	6.710 [5.610, 8.560]	0.0358	0.0618
Lymphocyte count (10^9/L)	0.800 [0.435, 1.240]	1.380 [0.800, 1.915]	0.0003	0.0013
Neutrophil count (10^9/L)	7.810 [4.000, 11.040]	4.510 [3.390, 6.290]	0.0032	0.0100
Platelet count (10^9/L)	180.000 [108.000, 221.000]	218.000 [162.500, 262.000]	0.0411	0.0635
eGFR ( ml/min/1.73m2)	80.230 [56.700, 95.855]	85.210 [69.425, 96.850]	0.3452	0.3858
Creatinine (umol/L)	69.000 [56.000, 109.000]	71.000 [59.500, 78.500]	0.6720	0.6720
BUN (mmol/L)	8.400 [5.600, 13.350]	5.700 [4.650, 9.500]	0.0080	0.0197
Uric acid (umol/L)	226.000 [173.000, 335.000]	274.000 [203.500, 340.500]	0.0780	0.0927
TBIL (umol/L)	11.200 [8.200, 16.350]	10.100 [8.100, 14.550]	0.3940	0.4158
ALT (IU/L)	20.000 [14.000, 24.000]	22.000 [16.500, 27.000]	0.0672	0.0851
AST (IU/L)	27.000 [23.500, 34.000]	31.000 [26.000, 35.000]	0.0462	0.0635
PCT (ng/MI)	0.240 [0.090, 1.190]	0.040 [0.040, 0.175]	< 0.0001	0.0004
NLR	9.680 [3.765, 20.680]	3.160 [1.850, 6.415]	< 0.0001	0.0004
Patient age (year)	72.000 [65.500, 78.000]	68.000 [55.500, 72.500]	0.0083	0.0197

Normally distributed continuous variables: statistical description and t tests				
Variables	Positive (mean, sd)	Negative (mean, sd)	*P*-value	p.FDR (q)
RBC count (10^12/L)	3.168 (0.790)	3.773 (0.877)	0.0003	0.0005
Hemoglobin (Hb) (g/L)	97.570 (23.525)	114.256 (26.637)	0.0007	0.0010
TP (g/L)	59.949 (8.342)	65.974 (7.065)	0.0002	0.0005
Prealbumin (mg/L)	143.240 (68.409)	185.205 (63.391)	0.0017	0.0017

Statistical analysis of categorical variables by using Chi-square test				
Variables	Frequency(%)	X-squared	*P*-value	p.FDR (q)
	Triple infection (Yes, no)			
Patient Gender (F, M)	(32.9, 67), (43.5, 56.4)	0.866	0.3521	0.3521
Pleural effusion (Yes, No)	(43, 56.9), (25.6, 74.3)	2.676	0.1018	0.1272
Lung lobes involved (2, < 2)	(82.2, 17.7), (56.4, 43.5)	7.734	0.0054	0.0135

*Statistical significance: *P*<0.05

There were lower lymphocytes (CD3^+^ T cell, CD3^+^CD4^+^ T cells, CD3^+^CD8^+^ T cells, total lymphocytes), nutrition signature (TP and prealbumin), RBC, Hb and platelet in bacterial, fungal and viral triple infection group. However, there were higher CRP, PCT, neutrophils, NLR, and BUN in the triple infection group. In addition, the triple infection group showed a greater number of lobar infiltrates and longer hospital stays compared to the dual infection group

*Abbreviations:* *NLR *neutrophil-to-lymphocyte ratio, *Hb *hemoglobin, *eGFR *estimated glomerular filtration rate, *TBIL *total bilirubin, *TP *total protein, *CRP *C-reactive protein, *PCT *procalcitonin, *BUN *blood urea nitrogen

#### Age-stratified analysis

Among patients aged < 70 years, the viral-associated triple infection group exhibited significantly lower levels of CD3^+^ (446.5 vs. 978, q = 0.0048), CD3^+^CD4^+^ (223.5 vs. 568, q = 0.0045), CD3^+^CD8^+^ (213 vs. 351, q = 0.0239) T cells, lymphocyte count (0.7 vs. 1.4, q = 0.0045), TP (61 vs. 68, q = 0.0200), prealbumin, red blood cell count, and hemoglobin, as well as significantly higher levels of NLR (13.17 vs. 2.58, q = 0.0048), CRP (79.5 vs. 8, q = 0.0141), and D-dimer compared with the double infection group (Table 3). The triple infection group had numerically higher mortality and longer hospital stay, but these differences were not statistically significant after FDR correction.

In patients aged ≥ 70 years, similar trends were observed for all the above variables, but none reached statistical significance except for the number of lung lobes involved (OR = 6.5, 95% CI: 1.77–23.93, q = 0.0212).

**Table 3:** Age-stratified immune dysregulation in viral-associated triple infection

	< 70 years		≥ 70 years	
Continuous variables: statistical description and non-parametric tests (Mann-Whitney U analysis)				
variables	Triple vs. double (median)	*Q*-value	Triple vs. double (median)	*Q*-value
CD3 absolute count ( /U)	446.5 vs. 978	0.0048	631 vs. 720.5	0.1676
CD4 absolute count ( /U)	223.5 vs. 568	0.0045	284 vs. 434	0.0853
CD8 absolute count ( /U)	213 vs. 351	0.0239	256 vs. 306	0.4352
CRP (mg/L)	79.5 vs. 8	0.0141	32 vs. 30.25	0.8254
D-dimer (mg/L)	1.52 vs. 0.65	0.0098	0.87 vs. 0.69	0.9363
WBC count (10^9/L)	10.77 vs. 6.71	0.1858	8.35 vs. 6.56	0.1987
Lymphocyte count (10^9/L)	0.7 vs. 1.4	0.0045	0.86 vs. 0.93	0.4134
Neutrophil count (10^9/L)	9.71 vs. 4.51	0.0613	7.53 vs. 4.54	0.1009
Platelet count (10^9/L)	186.5 vs. 198	0.1872	172 vs. 230	0.3521
eGFR ( ml/min/1.73m2)	87.4 vs. 91.8	0.6301	78.4 vs. 82.5	0.8831
Creatinine (umol/L)	66.5 vs. 71	0.7448	73 vs. 70	0.9412
BUN (mmol/L)	9.15 vs. 5.4	0.0882	8.3 vs. 6.25	0.2539
Uric acid (umol/L)	254.5 vs. 312.7	0.4505	219 vs. 229	0.6466
TBIL (umol/L)	11.9 vs. 10.8	0.3831	10.5 vs. 9.4	0.7358
ALT (IU/L)	20 vs. 22	0.0613	20 vs. 19	0.9363
AST (IU/L)	26 vs. 31	0.1442	27 vs. 29.5	0.4352
PCT (ng/MI)	0.62 vs. 0.04	0.0013	0.14 vs. 0.04	0.1604
NLR	13.17 vs. 2.58	0.0048	8.19 vs. 4.42	0.1009
TP (g/L)	61 vs. 68	0.0200	59 vs. 64	0.1009
Prealbumin (mg/L)	139 vs. 201	0.0294	144 vs. 169	0.2974
RBC count (10^12/L)	3.01 vs. 4.27	0.0045	3.25 vs. 3.38	0.7361
Hemoglobin (HB) (g/L)	91.5 vs. 132	0.0045	98 vs. 98.5	0.8254
Hospital stay length	18 vs. 9	0.0822	22 vs. 11.5	0.2539

Statistical analysis of categorical variables by using Fisher’s exact test				
	OR (95%CI)	*Q*-value	OR (95%CI)	*Q*-value
Patient Gender (F)	0.85 (0.28–2.53)	0.7866	0.45 (0.14–1.45)	0.4584
Pleural effusion (Yes)	2.52 (0.76–8.4)	0.3184	1.76 (0.53–5.9)	0.5247
Lung lobes involved (2)	2.09 (0.66–6.62)	0.3289	6.5 (1.77–23.93)	0.0212
Mortality (Yes)	10.52 (1.25–88.45)	0.072	1.75 (0.34–9.13)	0.7113

*Statistial significance: *P* < 0.05

Age <70 years: Compared with the double infection group, the viral‑associated triple infection group showed significantly lower levels of multiple immune and nutritional markers and significantly higher levels of NLR, CRP, and D-dimer. Mortality and hospital stay length were higher but not significant after FDR correction. Age ≥70 years: Similar trends were observed for the above variables, but none were statistically significant except for the number of lung lobes involved

*Abbreviations:* *NLR *neutrophil-to-lymphocyte ratio, *Hb *hemoglobin, *eGFR *estimated glomerular filtration rate, *TBIL *total bilirubin, *TP *total protein, *CRP *C-reactive protein, *PCT *procalcitonin, *BUN *blood urea nitrogen

## Discussion

This study found over 83% of patients were aged 60 or older, highlighting the high incidence of coinfections in the elderly. This aligns with existing evidence, as Hong et al. reported that co-infection risk increases with age [14]. This study developed a mortality prediction model. Preliminary evidence suggests that the model is promising.

**Fig. 3:**
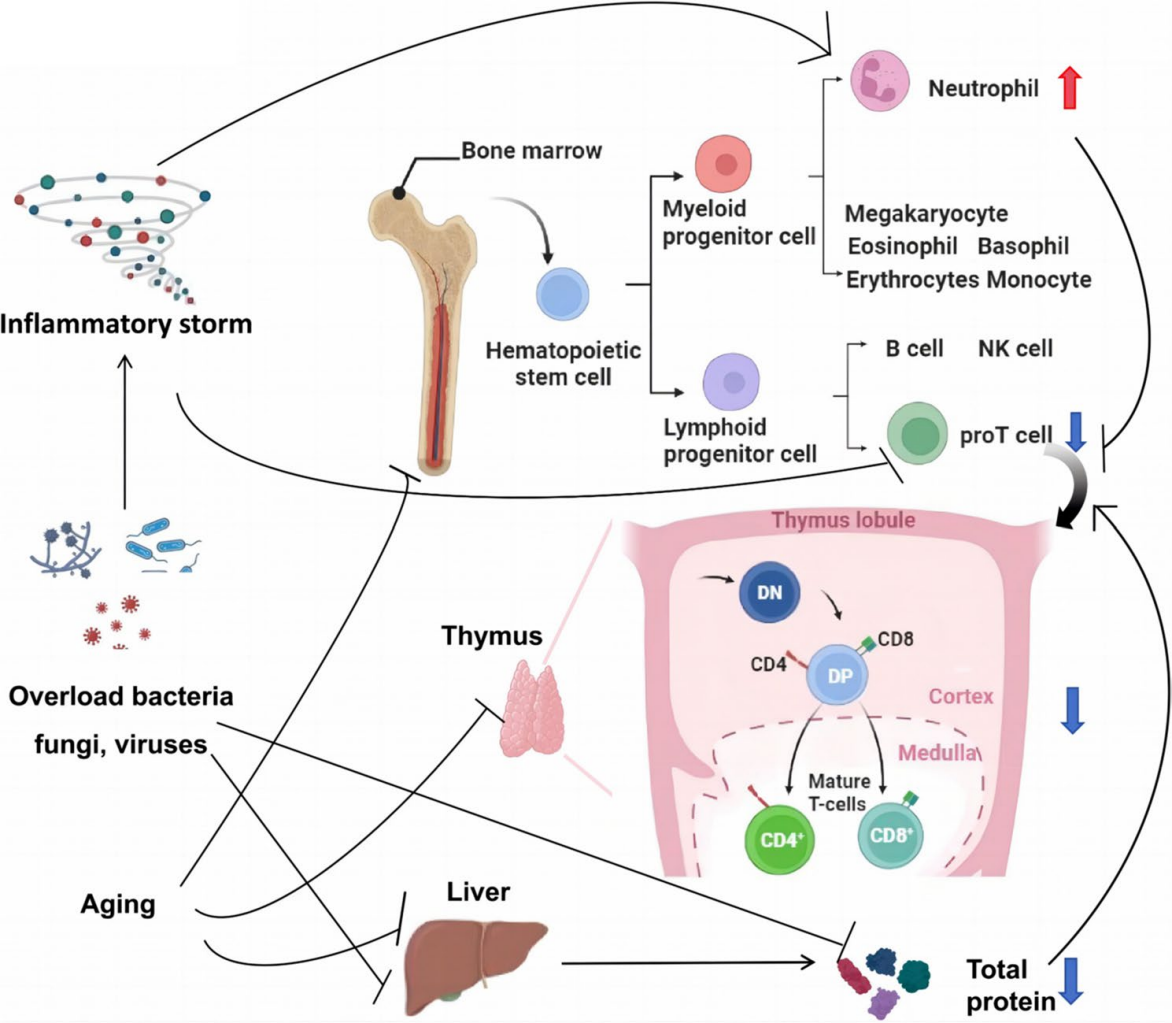
Mechanisms of immune dysregulation in the elderly: preserved myelopoiesis versus impaired lymphopoiesis. In the elderly, neutrophil production remains intact because myelopoiesis occurs entirely in the bone marrow, which is relatively preserved. Inflammatory factors can even boost neutrophil production. In contrast, T cell production can be impaired due to age-related thymic atrophy, affecting both CD3^+^CD4^+^ and CD3^+^CD8^+^ T cells. Inflammatory cytokines can further suppress lymphopoiesis. Additionally, declining liver function reduces TP synthesis, and inflammation consumes large amounts of albumin and globulins, while also inhibiting lymphocyte production. Together, these factors lead to a marked decrease in lymphocytes

Four key factors contribute to poor outcome: hyperinflammation, lymphopenia, polymicrobial infection, and aging (Fig. 3). In this context, NLR has been identified as an independent risk factor for mortality. Among deceased patients, the majority exhibited markedly elevated neutrophil counts (neutrophilia), accompanied by profoundly low lymphocyte (e.g., CD3^+^CD4^+^, CD3^+^CD8^+^ T cells) counts (lymphopenia). This pattern reflects the simultaneous occurrence of hyperinflammation and immunosuppression, two seemingly opposing yet coexisting immunological processes. In elderly patients facing multiple severe infections, the body enters a state of physiological emergency or stress, giving rise to the concurrent presence of both conditions [15].

In this study, hyperinflammation was characterized by increased neutrophils. Based on published literature, the possible mechanisms are as follows. Neutrophils originate from myeloid progenitor cells derived from pluripotent hematopoietic stem cells in the bone marrow [16]. As part of the innate immune system, they serve as the body’s “rapid response force” against pathogens [16]. When pathogens such as bacteria invade, the bone marrow is rapidly mobilized to produce and release neutrophils into the bloodstream [16]. Bacterial components and inflammatory factors (e.g., G-CSF, TNF-α) stimulate the bone marrow, triggering the rapid release of mature neutrophils from its reserve pool and ramping up their production [17]. The occurrence of neutrophilia suggests that bone marrow function remains preserved [17].

In the later stages of infection or in critically ill patients, the immune system may shift from an initial state of hyperactivation, characterized by neutrophilia, to a state of immune paralysis or immunosuppression [18, 19]. At this stage, lymphocyte counts decrease significantly (lymphopenia), and although neutrophil counts may remain elevated or fluctuate, their function can become impaired [19]. Patients in this state often struggle to clear pathogens, face a high risk of secondary infections, and subsequently experience increased mortality [20].

Notably, in this study, these patients exhibited lymphopenia, contrasting with the typical lymphocyte rise seen in viral infections [21]. Lymphopenia indicated immunosuppression, with a corresponding severe decline in TP (Fig. 3). Immunosenescence involves weakened responses to pathogens, alongside chronic inflammation [22]. T cells are key components of the adaptive immune system. They originate from the bone marrow and develop in the thymus. For the geriatric, age-related thymic involution impairs T-cell production and reduces pathogen resistance [20, 21]. As summarized by Mittelbrunn et al., T-cell aging includes a shrinking TCR repertoire, naïve-memory imbalance, and cellular senescence [23].

The severe infection or hyperinflammation aggravates the reduction in lymphocyte counts, resulting in lymphopenia [24]. Three other interconnected mechanisms explain this decline. First, excessive inflammatory factors (such as glucocorticoids and TNF-α) induce programmed cell death in lymphocytes [25]. Second, persistent antigenic stimulation drives lymphocytes into a state of exhaustion, characterized by impaired function and reduced proliferative capacity, further depleting their numbers [25]. Third, under the influence of inflammatory chemokines, lymphocytes exit the bloodstream and migrate to infected tissues or organs (e.g., the lungs) to fight pathogens, leading to a decrease in detectable peripheral blood counts [25]. This impaired response is primarily attributed to declined immune function and a heightened inflammatory state in the elderly [26].

Total protein includes albumin and globulin. Serum albumin (SA) can bind various inflammatory mediators and participate in regulating immune responses during systemic inflammation [27]. The conformational state of albumin can influence the mechanical properties of erythrocytes [28]. Globulins include antibodies, complement proteins, cytokines, and acute phase proteins [29, 30]. While TP is often dismissed as a mere nutritional marker, it is actually associated with humoral immunity, complement activity, and metabolic reserve [30]. The nutrient-sensing system is dysregulated in the elderly [31]. Elevated inflammation and oxidants accelerate the loss of TP [32]. Hence, an excessive reduction in TP can lead to immunosenescence.

RBC count and Hb concentration were significantly lower than in the viral-negative group. They function not only in oxygen transport but also in pathogen defense and innate immune regulation [33]. During infection, pathogens may acquire iron from Hb to enhance virulence; conversely, bacterial proteases trigger the activation of Hb, which in turn produces reactive oxygen species (ROS) that kill bacteria [34].

In this research, when elderly patients with bacterial and fungal co-infection acquired a viral infection, a significant decrease in lymphocytes, TP, and Hb, along with an increased NLR, was observed. When different pathogens (viruses, bacteria, fungi) infect the same host cell, they can actively assist each other in disrupting epithelial barriers and evading the immune system by binding with each other [35, 36]. In addition, high-level pathogenic stimulation induces T-cells to express multiple inhibitory receptors (like PD-1, TIGIT) [37]. When a virus infects an organism burdened with senescent cells, it triggers a dangerously amplified response [38]. The virus causes an accumulation of reactive oxygen species in these senescent cells [39]. This, in turn, reduces the expression of a protein called PDLIM2, which normally acts as a brake on the powerful inflammatory pathway NF-κB [39].

This study has limitations inherent to its single-center, retrospective design, as well as the lack of external validation. This model remains a preliminary, clinically interpretable prediction tool showing promising discrimination and calibration only in this single-center cohort. To enhance reliability, rigorous subject selection and robust statistical methods were employed. Bootstrap internal validation was performed to partially address overfitting concerns. With only 23 events (EPV < 10), we applied elastic net and bootstrap internal validation to partially address overfitting concerns. Bootstrap analysis revealed optimism in performance metrics, which internal methods cannot fully correct. Therefore, larger-sample external validation and prospective multicenter studies are essential to confirm the model’s generalizability and clinical utility before any implementation.

## Conclusion

This study reveals the paradoxical coexistence of hyperinflammation and lymphopenia in the study population. The majority of deceased patients were characterized by profound lymphopenia with neutrophilia and severe hypoproteinemia, reflecting immune dysregulation. A simple, clinically interpretable prediction model incorporating TP, NLR, patient age, and viral-associated triple infection was established to predict mortality. Patient age and viral-associated triple infection were forced into the final model as mandatory confounders to ensure unbiased estimation. The model showed promising discrimination and calibration in this single-center retrospective cohort. However, given the inherent limitations of the single-center, retrospective design and the lack of external validation, the model should be considered preliminary. Larger-sample external validation, ideally through prospective multicenter studies, is essential to confirm its generalizability and clinical utility before any implementation.

## Supplementary Information

Supplementary Material 1.

## Data Availability

Due to ethical restrictions and participant confidentiality agreements, the data that support the findings of this study are not publicly available. They are available on reasonable request from the corresponding author, professor Zhihong Xu, subject to approval by the Ethics Committee of Shanghai Jiao Tong University Affiliated Ruijin Hospital and the execution of a data use agreement.
