# Supplementary material for: The controversial interplay between hyperinflammation and immunosenescence in elderly critically ill co-infected patients
Source: BMC Geriatr. 2026 Jun 5;26:954. doi: 10.1186/s12877-026-07767-2 (PMC13366926; doi:10.1186/s12877-026-07767-2)
Supplement: Supplementary file 1 — Supplementary Material 1. [file 12877_2026_7767_MOESM1_ESM.docx]

| Supplementary table 1 Normality testing of numerical variables (method: Shapiro-Wilk). | | | | | |
| --- | --- | --- | --- | --- | --- |
| Virus Infection Status | Positive |  |  | Negative |  |
| Variables | W | P-value |  | W | P-value |
| CD3 absolute count ( /U) | 0.6116 | <0.0001 |  | 0.8277 | <0.0001 |
| CD4 absolute count ( /U) | 0.4658 | <0.0001 |  | 0.8521 | 0.0001 |
| CD8 absolute count ( /U) | 0.8326 | <0.0001 |  | 0.7518 | <0.0001 |
| CRP (mg/L) | 0.8641 | <0.0001 |  | 0.7233 | <0.0001 |
| D-dimer (mg/L) | 0.9533 | 0.0058 |  | 0.8758 | 0.0005 |
| WBC count (10^9/L) | 0.8585 | <0.0001 |  | 0.6215 | <0.0001 |
| Lymphocyte count (10^9/L) | 0.6208 | <0.0001 |  | 0.8793 | 0.0006 |
| Neutrophil count (10^9/L) | 0.8409 | <0.0001 |  | 0.6017 | <0.0001 |
| NLR | 0.4241 | <0.0001 |  | 0.4331 | <0.0001 |
| RBC count (10^12/L) | 0.9796 | 0.2380 |  | 0.9467 | 0.0636 |
| HB (g/L) | 0.9788 | 0.2127 |  | 0.9467 | 0.0636 |
| Platelet count (10^9/L) | 0.8988 | <0.0001 |  | 0.9842 | 0.8492 |
| eGFR ( ml/min/1.73m2) | 0.9533 | 0.0057 |  | 0.9581 | 0.1536 |
| Creatinine (umol/L) | 0.5954 | <0.0001 |  | 0.7948 | <0.0001 |
| BUN (mmol/L) | 0.7794 | <0.0001 |  | 0.5987 | <0.0001 |
| Uric acid (umol/L) | 0.9471 | 0.0025 |  | 0.9528 | 0.1018 |
| TBIL (umol/L) | 0.2955 | <0.0001 |  | 0.5103 | <0.0001 |
| TP (g/L) | 0.9874 | 0.6350 |  | 0.9577 | 0.1489 |
| Prealbumin (mg/L) | 0.9776 | 0.1788 |  | 0.9644 | 0.2501 |
| ALT (IU/L) | 0.9428 | 0.0015 |  | 0.9585 | 0.1588 |
| AST (IU/L) | 0.7648 | <0.0001 |  | 0.9562 | 0.1325 |
| PCT (ng/MI) | 0.2767 | <0.0001 |  | 0.1834 | <0.0001 |
| Patient age (year) | 0.8916 | <0.0001 |  | 0.9314 | 0.0199 |
| * Normal distribution: P>0.05 | | | | | |

Supplementary table 1 Normal distribution assessment was performed using the Shapiro-Wilk test on 23 numerical variables across two groups: triple co-infection and double co-infection. W: test statistic. A p-value >= 0.05 was used as the threshold to indicate normality. The RBC count, HB, TP, and prealbumin in both groups all follow a normal distribution. The other variables in both groups deviated significantly from normality (p < 0.05). NLR: Neutrophil-to-lymphocyte ratio. HB: Hemoglobine. GFR: Estimated glomerular filtration rate. TBIL: Total bilirubin. TP: Total protein.
